# Lipids and atrial fibrillation: New insights into a paradox

**DOI:** 10.1371/journal.pmed.1004067

**Published:** 2022-08-11

**Authors:** Dimitrios Sagris, Stephanie L. Harrison, Gregory Y. H. Lip

**Affiliations:** 1 Liverpool Centre for Cardiovascular Science, University of Liverpool and Liverpool Heart & Chest Hospital, Liverpool, United Kingdom; 2 Department of Internal Medicine, Faculty of Medicine, School of Health Sciences, University of Thessaly, Larissa, Greece; 3 Department of Cardiovascular and Metabolic Medicine, Institute of Life Course and Medical Sciences, University of Liverpool, Liverpool, United Kingdom; 4 Department of Clinical Medicine, Aalborg University, Aalborg, Denmark

## Abstract

In this Perspective, Dimitrios Sagris, Stephanie Harrison, and Gregory Lip discuss new evidence concerning the paradoxical relationship between circulating lipids and atrial fibrillation.

Although the prevalence of cardiovascular comorbidities and associated risk factors such as diabetes mellitus, chronic kidney disease, and obesity increase with age, lipid levels may go through several changes over the course of a lifetime, associated with sex, ethnicity, and metabolic profile [1]. The association between lipid levels and atherosclerotic disease is well established [2], but the association between lipid levels and incidence of atrial fibrillation (AF) has not been fully elucidated.

Despite the association between high lipoprotein levels and the increased risk of atherosclerosis and coronary artery disease (CAD), which, in turn, may lead to an increased risk of AF [3], several studies have suggested that high levels of low-density lipoprotein cholesterol (LDL-C), total cholesterol (TC), and high-density lipoprotein cholesterol (HDL-C) are associated with a lower risk of AF [4]. The clinical significance and pathophysiological mechanisms of this paradoxical inverse association between lipid levels and AF risk remain unclear [4].

In an accompanying study in *PLOS Medicine*, Mozhu Ding and colleagues conducted a large population-based study of >65,000 adults aged 45 to 60 years without any history of cardiovascular disease, using data from the Swedish National Patient Register and Cause of Death Register [5]. Using International Classification of Diseases (ICD) codes from discharge diagnoses of hospital visits and causes of death captured in these registries, participants were followed up for up to 35 years for incident AF. Higher levels of TC and LDL-C were associated with lower risk of AF within the first 5 years (hazard ratios [HRs]: 0.61, 95% confidence intervals [CIs]: 0.41 to –0.99; HR: 0.64, 95% CI: 0.45 to 0.92), but the effect was attenuated after 5 years of follow-up. Conversely, lower levels of HDL-C, high triglyceride (TG) levels and high TG/HDL-C ratio were consistently associated with a higher risk of AF over 3 decades of follow-up (HRs ranging from 1.13 [95% CI: 1.07 to 1.19, *p* < 0.001] to 1.53 [95% CI: 1.12 to 2.00]).

Previous longitudinal studies have demonstrated that levels of the majority of lipoproteins increase significantly between the ages of 20 to 50 years before plateauing in older age; this pattern is observed mainly in men, while in women, increasing lipoproteins are associated with menopause [6]. These early findings were recently confirmed in a cross-sectional study in a Chinese population, in which TC and LDL-C were found to plateau between the ages of 40 and 60 in men and 60 years of age in women, before declining markedly [7]. Considering the inverse association of TC and LDL-C with aging, and the association between aging and higher prevalence of AF, this may partially explain the inverse association of TC and LDL-C with AF.

Another interesting finding reported by Mozhu Ding and colleagues is the inverse association of HDL-C levels with AF incidence and the association of high TG levels and high TG/HDL-C ratio with increased risk of AF [8]. This association remained consistent for more than 10 years, suggesting a potentially strong association of HDL-C and TG with incident AF. Since HDL-C and high TG levels are important components of metabolic syndrome, this finding may demonstrate a role of metabolic syndrome and its components in the risk of AF. A recent meta-analysis of 6 cohort studies, including 30,810,460 patients, showed that metabolic syndrome and low HDL-C were associated with a significantly higher risk of AF (HR: 1.57; 95% CI: 1.40 to 1.77, and HR: 1.18; 95% CI: 1.06 to 1.32, respectively) [9]. Based on this evidence, we can speculate that a combination of low HDL-C and high non-HDL-C (i.e., TC excluding HDL-C) may have a potential association with AF risk. Nonetheless, a nationwide cross-sectional survey suggested that non-HDL-C may also be associated with a lower risk of AF [10].

The associations observed in the study by Mozhu Ding and colleagues may have been related to lipid treatment therapies, but as the authors highlight, lipid-lowering medicines were uncommonly used in the first few years of the study period and it is unlikely that these could have influenced the associations observed at the beginning of the follow-up period.

The association of lipid levels with incident AF remained consistent both in patients with and without heart failure (HF) or CAD. However, in the sensitivity analysis including only patients for whom data on use of medications were available, among those with HF or CAD being treated with lipid-lowering medication, the risk of AF was lower compared to those who were not treated [8]. It seems that although lipid levels are correlated with incident AF irrespective of the presence of HF or CAD, in this high-risk population, the use of lipid-lowering medication reduces the risk of AF, as was previously suggested [11].

Mozhu Ding and colleagues have been able to conduct a large-scale study with a long follow-up period, and the findings agree with previous observational evidence. As with all observational studies, residual confounding may be present. In this study, baseline lipid levels were assessed, but variability over time was not examined. A previous nationwide study in Korea has suggested high variability in lipid levels is associated with a higher risk of AF development [12]. Additionally, smoking or physical activity, which represent important cardiovascular parameters, were not accounted for, which may have partially influenced the observed associations.

Although the natural progression of vascular aging, chronic inflammation, and dynamic changes in cardiovascular risk factors, including dyslipidemia, may play an essential role in cardiovascular diseases and the risk of AF [13,14], the exact mechanisms of the potential inverse correlation of hyperlipidemia to AF remain elusive. The accompanying study of Mozhu Ding and colleagues supports the existing evidence on the paradoxical inverse correlation of TC, LDL-C, and HDL-C levels with the risk of future AF, providing further insights in the role of TG levels and their correlation to HDL-C levels. New insights may improve understanding of the pathophysiology behind this paradoxical observation [15]. Until then, hyperlipidemia should be assessed as part of the overall cardiovascular risk [16], and the AF paradox should not outweigh this risk.

## Abbreviations:

AF: atrial fibrillation
CAD: coronary artery disease
CI: confidence interval
HDL-C: high-density lipoprotein cholesterol
HF: heart failure
HR: hazard ratio
ICD: International Classification of Diseases
LDL-C: low-density lipoprotein cholesterol
TC: total cholesterol
TG: triglyceride
